# FBXO32 links ubiquitination to epigenetic reprograming of melanoma cells

**DOI:** 10.1038/s41418-020-00710-x

**Published:** 2021-01-18

**Authors:** Nadia Habel, Najla El-Hachem, Frédéric Soysouvanh, Hanene Hadhiri-Bzioueche, Serena Giuliano, Sophie Nguyen, Pavel Horák, Anne-Sophie Gay, Delphine Debayle, Nicolas Nottet, Guillaume Béranger, Brigitte Bressac-de Paillerets, Corine Bertolotto, Robert Ballotti

**Affiliations:** 1 Université Nice Côte d’Azur, Nice, France; 2 Inserm U1065, C3M, Team 1, Biology and Pathologies of Melanocytes, Equipe labellisée ARC 2019, Nice, France; 3 CNRS, Institut de Pharmacologie Moléculaire et Cellulaire, Sophia Antipolis, France; 4 Département de Biopathologie et INSERM U1279, Gustave Roussy, Villejuif, France

**Keywords:** Oncogenes, Epigenetics

## Abstract

Ubiquitination by serving as a major degradation signal of proteins, but also by controlling protein functioning and localization, plays critical roles in most key cellular processes. Here, we show that MITF, the master transcription factor in melanocytes, controls ubiquitination in melanoma cells. We identified FBXO32, a component of the SCF E3 ligase complex as a new MITF target gene. FBXO32 favors melanoma cell migration, proliferation, and tumor development in vivo. Transcriptomic analysis shows that FBXO32 knockdown induces a global change in melanoma gene expression profile. These include the inhibition of CDK6 in agreement with an inhibition of cell proliferation and invasion upon FBXO32 silencing. Furthermore, proteomic analysis identifies SMARC4, a component of the chromatin remodeling complexes BAF/PBAF, as a FBXO32 partner. FBXO32 and SMARCA4 co-localize at loci regulated by FBXO32, such as CDK6 suggesting that FBXO32 controls transcription through the regulation of chromatin remodeling complex activity. FBXO32 and SMARCA4 are the components of a molecular cascade, linking MITF to epigenetics, in melanoma cells.

## Introduction

Ubiquitination is a post-translational modification initially described to play a key role in protein homeostasis through subsequent degradation of targeted proteins by proteasome or lysosomes. Ubiquitination was also shown to regulate protein interaction, functioning, and localization. The specificity of the effects of ubiquitination is controlled by a complex ubiquitin code [1]. Ubiquitination controls a wide spectrum of cellular processes that includes NF-κB pathway activation, DNA damage repair, cell death, autophagy, or metabolism [2]. Therefore, it is not surprising that dysregulation of ubiquitination process has broad consequences in cellular biology, leading to numerous pathologies, including cancer. The characterization of the regulation of genes involved in ubiquitination, as well as the cellular processes regulated by ubiquitination are therefore of paramount importance to improve our knowledge on the associated diseases.

Cutaneous melanoma arises from melanocytes, neural crest-derived cells producing skin pigments. It is the most aggressive forms of skin cancer because of its high propensity to metastasize. Metastatic melanoma is the leading cause of skin cancer-related mortality. In the last decade, stunning improvement of metastatic melanoma treatment has been achieved by using therapies targeting *BRAF*^*V600E*^, the most frequent somatic mutation in melanoma and most recently, with immunotherapies targeting the negative immune check points CTLA4 and PD1. Despite a huge response rate, the targeted therapies did not reach the initial expectations because of quasi systematic relapses, ensuing resistance acquisition. With immunotherapies, up to 40% of long-term responders have been described, but most of the patients are or become resistant to these therapies. Resistance can arise from genetic alterations, among which new mutations in *NRAS* or *MEK* for resistance to targeted therapies, and in *JAK* or *B2M* for resistance to immunotherapies [3].

In addition, melanoma cells are highly plastic, and resistance is also frequently caused by a switch from proliferative to invasive phenotype, ensuing epigenetic and transcriptional rewiring [4, 5].

Compelling data have been gathered showing the involvement of ubiquitination in melanoma development [6]. Among them, the deubiquitinase, BRCA-1-associated protein-1 is frequently mutated in uveal melanomas [7] and in a subset of cutaneous melanomas [8], where it functions as a tumor suppressor. FBXW7, a F-Box/WD repeat containing protein that constitutes a subunit of the ubiquitin protein ligase complex, SKP1-cullin-F-box (SCF), and PARKIN, an E3 ubiquitin ligase, also function as tumor suppressors and were found mutated in melanomas [9, 10]. More recently, HACE1, HECT domain and ankyrin repeat-containing E3 ubiquitin protein ligase that regulates RAC1 activity, was also reported to favor melanoma invasiveness [11].

Microphthalmia-associated transcription factor (MITF), the key transcription factor in melanocytes, is also known to play a crucial role in melanoma phenotypic switch [12–14] and therapy resistance [15–17].

MITF was initially described to control differentiation and pigmentation through the regulation of genes involved in melanogenesis [18], then MITF was also involved in melanoma cell survival and proliferation, while it represses motility and invasive capacities. The involvement of MITF in such a large array of biological processes makes of MITF a crucial player in melanoma development [19].

In this work, we sought to investigate the role of MITF in the ubiquitination processes in melanoma cells. We identified at least three distinct genes regulated by MITF that are also involved in the ubiquitination process. Among them, we focused our attention on *FBXO32* because it is located on chromosome 8q, a region frequently amplified in melanoma and its role in melanomas has never been studied. We performed a comprehensive analysis of the role of *FBXO32* in melanoma, showing that it regulates key biological processes and transcriptional programs of melanoma cells through epigenetics mechanisms.

## Materiel and methods

### Cell cultures and reagents

Human melanoma cell lines A375, MeWo, and SKMel 28 were from ATCC. 501Mel cell line was provided by Colin Goding (Oxford). All cell lines were used less than ten passages after STR profiling. Cells were cultured in Dulbecco’s Modified Eagle’s medium supplemented with 7% fetal bovine serum (FBS) and 1% penicillin-streptomycin. short-term cultures were isolated from metastatic malignant melanoma fresh sterile tissues obtained from the Nice CHU Hospital. Written informed consent was obtained from each patient included in this study, and the study was approved by the hospital ethics committee (Nice Hospital Center and University of Nice Sophia Antipolis, No. 210-2998). The study was performed in accordance with the Declaration of Helsinki. Cells were cultured in RPMI medium supplemented with 10% FBS and 1% penicillin-streptomycin. Cells were grown at 37 °C in a humidified atmosphere containing 5% CO_2_. Melanocytes were obtained from children foreskin (5- and 7-year old) by overnight digestion in phosphate-buffered saline (PBS) containing 0.5% dispase grade II at 4 °C, followed by a 1-h digestion with trypsin/EDTA solution (0.05%:0.02% in PBS) at 37 °C. Cells were grown in MCDB 153 medium supplemented with FCS 2%, 0.4 μg/ml hydrocortisone, 5 μg/ml insulin, 16 nM PMA, 1 ng/ml b-FGF, and penicillin/streptomycin (100 U/ml:50 μg/ml).

FBXO32 lentivirus construction (RC223661L1) was from Origen.

### Transient transfection of siRNA

Briefly, a single pulse of 25 nM of siRNA was administered to the cells at 50% confluency through transfection with 5 µl of Lipofectamine^TM^ RNAiMAX in Opti-MEM medium (Invitrogen). siRNA-mediated downregulation of MITF was achieved with specific sequences 5′-GGUGAAUCGGAUCAUCAAGTT-3′ and 5′-CUUGAUGAUCCGAUUCACCTT-3′ from Invitrogen. FBXO32 (SI04366166, SI04317803), CDK6 (SI00024360, SI00605052), and SMAD7 (SI00082537, SI00082544) siRNAs were purchased from Qiagen.

### Cell migration

Cell migration was carried out using a Boyden chamber assay with 8 µm pore filter inserts (BD Bioscience). Cells (100 × 10^3^) were seeded on the upper chamber of a trans-well and RPMI + 10% FBS placed into the lower chamber. Sixteen hours later, adherent cells to the underside of the filters were fixed with 4% paraformaldehyde, stained with 0.4% crystal violet, and counted. Results represent the average of triplicate samples from three independent experiments.

### Cell proliferation

The cells were seeded onto 12-well dishes (1 × 10^4^ cells), and at 48 h post-transfection, they were detached with trypsin from day 1 to 4 and counted in triplicate using a hemocytometer. The experiments were performed at least three times.

### Colony formation assay

Human melanoma cells were seeded onto 6-well plates. The cells were subsequently placed in a 37 °C, 5% CO_2_ incubator. Colonies were grown before being stained with 0.04% crystal violet/2% ethanol in PBS for 30 min. Photographs of the stained colonies were captured. The colony formation assay was performed in duplicate.

### Western blot

Cytoplasmic fraction, nuclei fraction, and total cell lysates were separated by SDS-PAGE, transferred onto a polyvinylidene difluoride membrane and then probed with antibodies to FBXO32 (Abcam, Ab168372), MITF (Abcam, Ab12039), ubiquitin (Abcam, ab7254), CDK6 (DCS83, Cell Signaling Technology), SMAD7 (Santa Cruz Biotechnology, sc-11392), BRG1 (Abcam, ab10641), Baf60a (Santa Cruz Biotechnology, sc-51440), and HSP90 (Santa Cruz Biotechnology, sc-13119). Horseradish peroxidase-conjugated anti-rabbit or anti-mouse antibodies were from Dako. Proteins were visualized with the ECL system (Amersham).

### mRNA preparation and real-time/quantitative PCR

mRNA isolation was carried out with TRIzol (Invitrogen) according to standard procedure. QRT-PCR was performed using SYBR^®^ Green I (Eurogentec, Seraing, Belgium) and Multiscribe Reverse Transcriptase (Applied Biosystems) and subsequently monitored by the ABI Prism 7900 Sequence Detection System (Applied Biosystems, Foster City, CA). Primer sequences for each cDNA were designed using either Primer Express Software (Applied Biosystems) or qPrimer depot (http://primerdepot.nci.nih.gov) and are available upon request.

Detection of RPL0 gene was used to normalize the results. Primer sequences for each cDNA were designed using either Primer Express Software (Applied Biosystems) or qPrimer depot (http://primerdepot.nci.nih.gov), and these sequences are available upon request.

### Proteomics analysis and nano-HPLC-MALDI-TOF/TOF analysis

Proteins from parental or MYC/DDK-FBXO32-expressing 501Mel melanoma cells were extracted in buffer containing TRIS-HCl pH7.5 50 mM, NaCl 15 mM, Triton X-100 1%, supplemented with protease and phosphatase inhibitors. Cell lysates (2 mg) were immunoprecipitated with anti-DDK antibody, washed and eluted with DDK peptide. Then, samples were immunoprecipitated again with anti-Myc antibody and separated by SDS-PAGE.

Proteins contained into gel slices were reduced/alkylated and digested by a treatment with DTT/IAA and trypsin. Peptides extracted were separated using a nano-HPLC (250 mm column, Ultimate 3000, ThermoFisherScientific). Nano-HPLC was coupled to Q-exactive plus mass spectrometer (ThermoFisherScientific). Data were reprocessed using Proteome Discoverer 2.2 equipped with Sequest HT. Files were searched against the Swissprot Homo sapiens FASTA database. Two separate experiments were performed, and all the proteins detected in one of the two immunoprecipitations from parental cells were considered as “non-specific.”

### Chromatin immunoprecipitation (ChIP)

Cross-linked chromatin was prepared as previously described [20]. BRG1 ChIP was performed on 501Mel expressing or not Myc/DDK-tagged FBXO32 (5 × 10^7^ cells per condition), using BRG1 or DDK antibody or an isotype matched control immunoglobulin, and analyzed by real-time PCR. Data are expressed as a percentage of enrichment compared to control immunoglobulin. Forward and reverse real-time PCR primers used for the human genomic DNA analysis are as follows. CDK6: 5′-AAGAACGGAGGCCGTTTCGTG-3′; 5′-TTTCTGGGCCTGAGGATTCCC-3′ HDAC3: 5′-GTGCTGCGCAAGCACGTAGC-3′; 5′-CAAATGGCCCTCGCATCCTA-3′. As negative control, we used for CDK6, an intronic amplicon, chr7:92454544-92454710. CDK6neg 5′ TCCTTGCAGTATCCCAAGCAT 3′; 5′-GGTGAGGTCTCTGGCATTCAG 3′. For HDAC3, the negative control was an intronic amplicon chr5:141001678-141001852.HDAC3neg 5′ GAGTACCTGTTGGGCCCTG 3′; 5′ CCTGGATGTAGGTAAGGGCTAGC-3′. No enrichment with these primers was observed in DDK or BRG1 immunoprecipitates compared to Ctl antibody.

### Animal experimentation

Animal experiments were performed in accordance with French law and approved by a local institutional ethical committee (#NCE/2017-283). The animals were maintained in a temperature-controlled facility (22 °C) on a 12-h light/dark cycle and provided free access to food (standard laboratory chow diet from UAR, Epinay-S/Orge, France). Human MeWo, SKmel28, and A375 melanoma cells, infected with control (LV) or FBXO32-encoding lentiviral vectors, were subcutaneously inoculated into 8-week-old female, immune-deficient, athymic, nude FOXN1^*nu*^ mice (Harlan Laboratory). The growth tumor curves were determined after measuring the tumor volume (V) using the equation $V = \frac{{L \,\times\, W^2}}{2}$, where L is tumor length and W is tumor width. At the end of the experiment, the mice were euthanized by cervical dislocation, and the tumors were harvested for protein extraction.

### Immunofluorescence studies

Short-term culture derived from patient was grown on glass coverslip (2 × 10^4^ cells per point) in 12-well dishes. Cells were then washed, fixed at room temperature for 20 min with 4% paraformaldehyde (PFA, Sigma-Aldrich), and permeabilized by a 10-min treatment with 50 mM NH_4_Cl, BSA 3% in PBS followed with 2-min treatment with BSA 3%, 1% Triton X-100 in PBS, before being exposed to anti-FBXO32 or anti-BRG1 antibodies for 1 h at room temperature. Cells were finally incubated with appropriate secondary fluorescent-labeled antibodies (Invitrogen Molecular Probes) for 1 h at room temperature and mounted using Gel/Mount (Biomeda Corp., Foster City, CA). Immunofluorescence was examined and photographed using a Zeiss Axiophot microscope equipped with epifluorescence illumination.

### Gene expression profiling

Total RNAs from three different melanoma cell lines (501Mel, SKMel28, and WM9) and one short-term culture treated with control or FBXO32 siRNA were extracted using the RNeasy kit (Qiagen), according to the supplier’s recommendations. mRNA expression profiling was performed with 8 × 60 K high-density SurePrint G3 gene expression human Agilent microarray, in accordance with the protocol described by the manufacturer. Microarray data analyses were performed using R (http://www.r-project.org/). The quality control was performed using the Bioconductor package ArrayQualityMetrics and custom R scripts. Additional analyses were performed using Bioconductor package Limma. Briefly, data were normalized using the quantile method. Replicated probes were averaged after normalization and control probes were removed. Then we used a linear modeling approach to calculate log ratios, moderated *t*-statistics, and *P*-values. *P*-values were adjusted for multiple testing using the Benjamini and Hochberg method, which controls the false discovery rate.

### Statistical analysis

No statistical methods were used to determine sample size. Sample size was determined to be adequate based on the magnitude and consistency of measurable differences between groups. The data are presented as the means ± SD and analyzed using two-sided Student’s *t*-test with Prism or Microsoft Excel software. For xenograft studies, sample size was determined using ClinCalc assuming a standard deviation of 30% in the control group and 50% of difference in FBXO32 group (*α* = 0.05, *β* = 0.1). No randomization was used, and no blinding was done. The data were analyzed using the non-parametric Mann–Whitney test. The difference between conditions was statistically significant at *p* < 0.05.

## Results

### MITF controls ubiquitination in melanoma cells

**Fig. 1:**
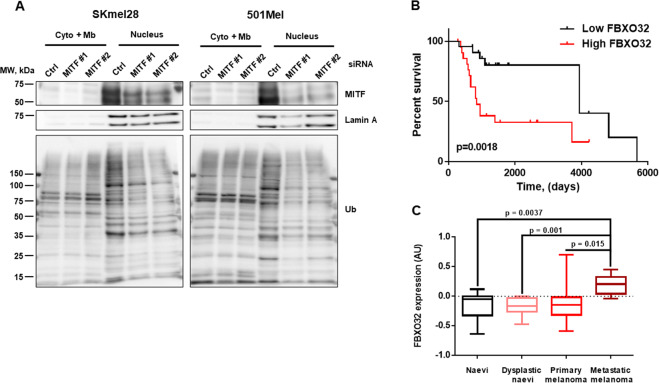
MITF controls ubiquitination in melanoma cells. **A** Western blot analysis showing the effect of MITF downregulation on ubiquitination in cytoplasmic/membrane and nuclear protein fractions in SKmel28 cells (left panel) and 501Mel cells (right panel). **B** Kaplan–Meier survival curve of cutaneous melanoma patients with low (black line) or high (red line) expression level of FBXO32 from GSE19234 dataset. **C** FBXO32 expression in human naevi (*n* = 18), dysplastic naevi (*n* = 11), primary melanomas (*n* = 23), and metastatic melanomas (*n* = 5) (GSE12391).

Studying the role of MITF in ubiquitination, in melanoma cells, we observed that MITF silencing, by two different siRNA, led to an inhibition of global ubiquitination in the nuclear fraction of human melanoma SKmel28 and 501Mel cell lines (Fig. 1A). This result indicated that MITF-regulated genes involved in ubiquitination processes. To uncover the mediators of MITF effect on ubiquitination, we interrogated a list of 780 genes (Gene Ontology, AmiGO2) involved in the ubiquitination processes. Among them, we selected those that (i) contained a ChIP-Seq-validated MITF binding site in their promoter [21], (ii) were correlated with MITF expression in the CCLE series of 61 melanoma cells (Log(2)FC > 1) (https://portals.broadinstitute.org/ccle/about), and (iii) were downregulated by siMITF (log(2)FC < –1).

After applying these stringent filters, three genes reached the threshold (Table 1). TRIM63, which belongs to the TRIpartite Motif protein family [22], and HERC5, a HECT domain E3 ligase, are endowed with a E3 ligase activity [23] and FBXO32, a F-box only protein is an essential component of the SCFs ubiquitin protein ligase complexes [24]. TRIM63 was already identified as a MITF target and was reported to be involved in melanoma cell migration and invasion [25]. We therefore did not pursue the investigation on TRIM63.

**Table 1:** List of genes involved in the ubiquitination process that are bound by MITF, correlated with MITF in the CCLE melanoma cell line panel, and inhibited by siMITF.

Chr no.	Distance to TSS	Max read	Gene name	Gene description	exp	Log FC MITF H/L	siMITF1	siMITF2
Chr 1	–476/–89	31/189	TRIM63	Tripartite motif containing 63, E3 ubiquitin protein ligase	6.78	5.27	–4.32	–4.34
Chr 4	–17	41	HERC5	HECT and RLD domain containing E3 ubiquitin protein ligase 5	7.00	2.00	–1.17	–1.07
Chr 8	–96	37	FBXO32	F-box protein 32	6.90	1.12	–1.39	–1.56

Further analysis of the TCGA melanoma cohort showed that HERC5 and FBXO32 expression covaried with MITF (Supplementary Fig. 1C, D); however, they were not correlated significantly with survival of patients with metastatic cutaneous melanoma (Supplementary Fig. 1A, B). Analysis of another cutaneous melanoma cohort (GSE19234) showed that patients with high FBXO32 expression had a decreased survival (Fig. 1B), whereas no significant association of HERC5 expression with survival (Supplementary Fig. 1E) was observed in the same cohort. Note that MITF expression is also associated with a poor survival in this cohort. This is not the case in the TCGA cohort.

Additional analysis of FBXO32 expression in the GSE12391 cohort, containing different stages of the disease, including nevi, dysplastic nevi, primary, and metastatic melanomas, showed that FBXO32 expression was increased in metastatic melanoma as compared to earlier stages (Fig. 1C).

Taken together these observations prompted us to investigate the role of FBXO32 in melanomas.

### FBXO32 is a MITF target

**Fig. 2:**
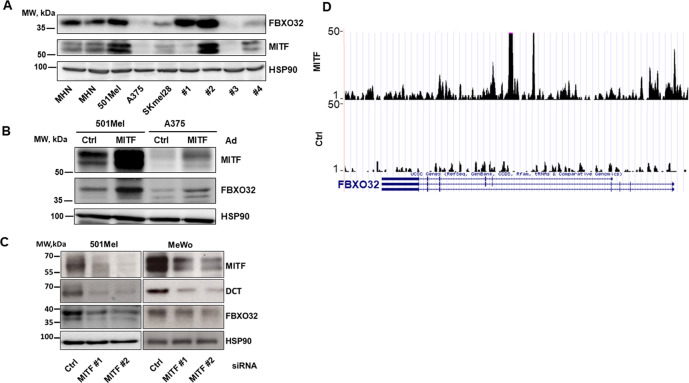
FBXO32 is a MITF target. **A** Western blot analysis of FBXO32 and MITF expression in normal human melanocytes (NHM), A375, 501Mel, SKmel28 cell lines, and in short-term culture of metastatic melanomas isolated from four different patients (1–4). **B** FBXO32 expression in 501Mel and A375 cells after adenovirus-mediated MITF forced expression. **C** FBXO32 and DCT expression in 501Mel and MeWo cells after siRNA-mediated MITF downregulation. HSP90 expression was probed as loading control. **D** UCSC image capture of the FBXO32 gene from MITF ChIP-Seq experiments [21] shows major MITF-binding sites.

First, we analyzed FBXO32 expression in three melanoma cell lines and four short-term cultures derived from patients’ biopsies in our laboratory. FBXO32 expression was variable and roughly followed the expression of MITF, except for cells isolated from patient 1 that expressed high level of FBXO32 but almost no MITF (Fig. 2A). Of note, these cells, isolated from a patient after treatment with BRAF inhibitor, are resistant to BRAF inhibitors. Then, we showed that adenovirus-forced MITF expression led to an increase in FBXO32 protein expression in both 501MEL and A375 melanoma cell lines (Fig. 2B), while MITF silencing decreased the expression of FBOX32 and DCT, a known transcriptional target of MITF in MeWo and 501Mel cell lines (Fig. 2C). Inhibition of FBXO32 expression ensuing MITF silencing was also verified by qPCR in 501Mel, MeWo, and two short-term melanoma cultures (Supplementary Fig. 2A–E). It should be noted that MITF silencing in cells from patient 1 did not result in a consistent inhibition of FBXO32 expression, further strengthening the lack of epistatic regulation of FBXO32 by MITF in these cells.

Finally, looking at the MITF ChIP-Seq data from Davidson’s lab [21], the UCSC image capture of the FBXO32 gene showed a binding of MITF at the FBXO32 promoter and in intronic region (Fig. 2D). Therefore, our data demonstrate an epistatic relation between MITF and FBXO32, the latter being a direct transcription target of MITF.

### FBXO32 controls migration of melanoma cells

**Fig. 3:**
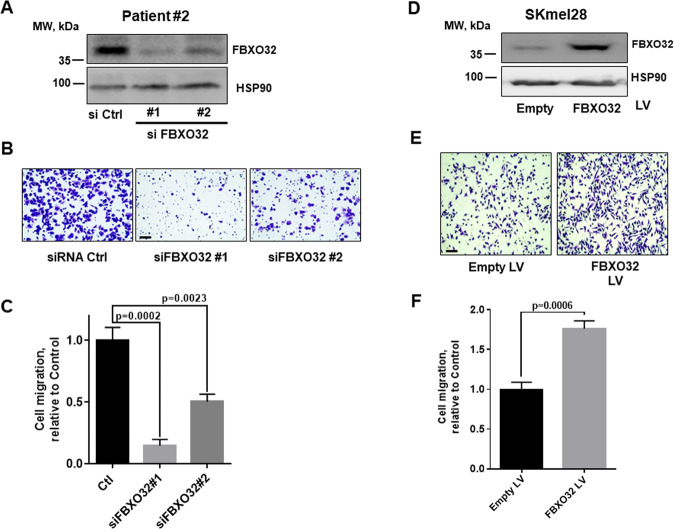
FBXO32 controls migration of melanoma cells. **A** Western blot analysis of FBXO32 expression after transfection of siRNAs targeting FBXO32 in melanoma cells isolated from patient 2. HSP90 expression was probed as loading control. **B** Representative image of the effects of FBXO32 downregulation on Boyden migration of melanoma cells from patient 2. Bar = 100 µm. **C** Quantification of patient 2 melanoma cell migration after FBXO32 downregulation (mean ratio ± SD, *n* = 3). **D** Western blot analysis of FBXO32 in SKmel28 cells after lentivirus-mediated transduction. HSP90 expression was probed as loading control. **E** Effects of FBXO32-forced expression on SKmel28 migration. Bar = 100 µm. **F** Quantification of SKmel28 migration after FBXO32-forced expression (mean ratio ± SD, *n* = 3).

In view to explore the biological functions of FBXO32 in melanomas, we studied the effect of FBXO32 silencing or forced expression on the motility of melanoma cells. Using two different siRNA targeting FBXO32, in melanoma cell expressing high endogenous FBXO32 (cells from patient 2), we observed that the inhibition of FBXO32 expression (Fig. 3A) led to a decrease in the migration of melanoma cells in Boyden chambers assays as demonstrated by the pictures of the lower face of the wells (Fig. 3B) and by quantification of three independent experiments (Fig. 3C). Similarly, the migration ability of 501Mel was reduced when FBXO32 expression was downregulated by doxycycline inducible specific shRNA (Supplementary Fig. 3A, B). Furthermore, the effect of FBXO32 silencing on migration cannot be ascribed to an inhibition of proliferation because after 16 h, the time used for migration evaluation, we did not observe relevant effect on 501Mel and SKmel28 proliferation, while the effect on migration is statistically significant (Supplementary Fig. 3C–J).

Conversely, forced FBXO32 expression using a lentiviral vector (Fig. 3D) stimulated the migration of SKmel28 melanoma cell line (Fig. 3E, F).

Taken together, these data demonstrate that FBXO32 enhances the migration of melanoma cells.

### FBXO32 controls proliferation and xenograft tumor development

**Fig. 4:**
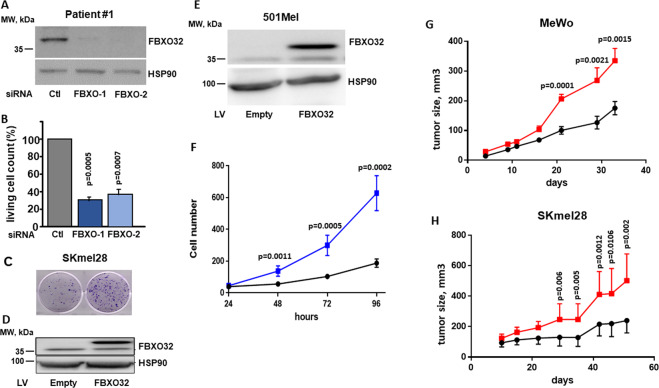
FBXO32 controls proliferation and xenograft development. **A** Western blot analysis of FBXO32 expression after transfection of siRNAs targeting FBXO32 in melanoma cells isolated from patient 1. HSP90 expression was probed as loading control. **B** Quantification of living cells 72 h after FBXO32 downregulation by siRNAs in melanoma cells from patient 1 (mean ratio ± SD, *n* = 3). **C** Colony formation assay of parental or FBXO32 overexpressing SKmel28 cells. **D** Western blot analysis of FBXO32 expression after FBXO32-forced expression in SKmel28 cells. HSP90 expression was probed as loading control. **E** Western blot analysis of FBXO32 expression after lentivirus-mediated FBXO32-forced expression in 501Mel cells. HSP90 expression was probed as loading control. **F** Quantification of 501Mel cell proliferation after empty virus (black line) or FBXO32 encoding virus (blue line) transduction, from 24 to 96 h (mean ± SD, *n* = 6). **G** Xenograft growth after subcutaneous injection of MeWo cells transduced with empty vector (black line) or FBXO32 encoding virus (red line) (mean ± SD, *n* = 10). **H** Xenograft growth after subcutaneous injection of SKmel28 cells transduced with empty vector (black line) or FBXO32 encoding virus (red line) (mean ± SD, *n* = 10).

In short-term culture from patient 1, FBXO32 knockdown by two different siRNA (Fig. 4A) inhibited cell proliferation (Fig. 4B). Conversely, forced expression of FBXO32 in SKmel28 cell line, by lentivirus infection (Fig. 4D), increased the formation of colonies (Fig. 4C). FBXO32-forced expression in 501Mel cell line (Fig. 4E) increased proliferation in vitro (Fig. 4F). FBXO32-forced expression also increased proliferation in A375 (Supplementary Fig. 4A, B) and SKmel28 cell lines expressing low level of endogenous FBXO32 (Supplementary Fig. 4C).

Furthermore, FBXO32-forced expression also favored the growth of MeWo and SKmel28 xenografts in nude mice (Fig. 4G, H). The same observations were made for xenografts with A375 cell line (Supplementary Fig. 4D). The increased expression of FBXO32 in the tumors was verified by western blot (Supplementary Fig. 4E). These data indicate that FBXO32 favors the proliferation of melanoma cells, both in vitro and in vivo.

### FBXO32 regulates the transcriptional program in melanoma cells

**Fig. 5:**
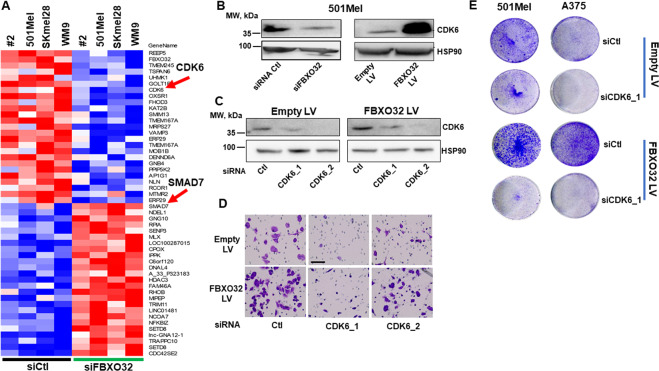
FBXO32 regulates the transcriptional program in melanoma cells. **A** Heat map showing the top 50 genes significantly affected by FBXO32 knockdown in melanoma cells from patient 2, 501Mel, SKmel28, and WM9 cell lines. **B** Western blot analysis of CDK6 expression after transfection of siRNAs targeting FBXO32 (left panel) or transduction by FBXO32-expressing lentivirus (right panel) in 501Mel cells. HSP90 was probed as loading control. **C** CDK6 expression after siRNAs transfection targeting CDK6 in 501Mel transduced with (right panel) or without (left panel) FBXO32 lentivirus. **D** Representative image of the effect of CDK6 downregulation on migration of 501Mel cells overexpressing (lower panel) or not (upper panel), FBXO32. Bar = 100 µm. **E** Effect of CDK6 downregulation on colony formation in 501Mel and A375 cells overexpressing, or not, FBXO32.

Then, we sought to gain insights on the molecular mechanisms involved in the modification of migration and proliferation induced through FBXO32 expression modulation. To do so, we performed a transcriptomic analysis in three different melanoma cell lines and one short-term culture (patient 2) treated with either control or FBXO32 siRNA. Statistical analysis (absLog2FC > 0.75, Adj. *p*-value < 0.05) identified 331 genes regulated upon FBXO32 knockdown, 216 downregulated, 115 upregulated (Supplementary Table 1). The heat map of the most differentially regulated genes (top 50) is shown (Fig. 5A). Ingenuity pathway analysis revealed that this gene list was associated with an inhibition of cell proliferation and migration (Table 2), in agreement with the proliferation and migration data obtained previously. IPA upstream activators analysis indicated that gene expression changes could result from MITF, MYC, or TGFβ pathway inhibition (Supplementary Table 2). The same analysis also predicted the activation of three microRNAs (mir145, mir124, Let7), p53, and KDM5B, a histone lysine demethylase, suggesting a link with epigenetics that is strengthened by the upregulation of HDAC3 in FBXO32 depleted cells.

**Table 2:** Ingenuity pathway analysis^®^ of the genes regulated upon FBXO32 silencing.

Diseases or functions annotation	*p*-value	*z*-score	Molecules
Proliferation of cells	3.39E–04	–3.66	104
Neoplasia of epithelial tissue	2.71E–04	–2.56	243
Cell proliferation of tumor cell lines	3.71E–03	–2.50	49
Cell proliferation of breast cancer cells	8.20E–03	–2.39	17
Epithelial cancer	4.34E–04	–2.36	241
Formation of cellular protrusions	7.73E–03	–2.35	28
Apoptosis of blood cells	5.98E–03	–2.27	18
Development of neurons	3.00E–04	–2.17	31
Invasion of tumor cell lines	6.19E–03	–2.16	24
Neuritogenesis	3.19E–04	–2.01	25

In agreement with the results obtained from gain- and loss-of-function experiments, upon FBXO32 silencing, CDK6 a protein kinase involved in cell proliferation was downregulated and SMAD7, an inhibitor of TGFβ signaling linked to cell migration, was upregulated.

Furthermore, western blot analysis in 501Mel cells infected with FBXO32-encoding lentivirus showed an increase in CDK6 expression, while FBXO32 knockdown led to its inhibition (Fig. 5B). Then, Boyden chamber experiments showed that CDK6 inhibition reduced migration in both parental and FBXO32 overexpressing 501Mel cells (Fig. 5C, D). The same observations have been made in A375 cells (Supplementary Fig. 5A, B). CDK6 knockdown also inhibited the proliferation of parental and FBXO32-overexpressing 501Mel and A375 cells (Fig. 5E).

We also validated the effect of FBXO32 on SMAD7 expression, by western blot showing increased SMAD7 level in cells treated with a siRNA to FBXO32 and a decreased SMAD7 level in cells with forced FBXO32 expression (Supplementary Fig. 6A). Furthermore, using TGFβ reporter assay, we demonstrated that the inhibition of SMAD7 expression, which was increased upon FBXO32 knockdown, prevented the inhibition of the TGFβ pathway in this condition, indicating that SMAD7 is a key player in the inhibition of the TGFβ pathway evoked by FBXO32 silencing (Supplementary Fig. 6B, C).

### FBXO32 is associated with chromatin remodeling complex at regulated loci

As FBXO32 is not a transcription factor, it remained to understand how it can modulate gene expression. To answer this question, we performed a tandem affinity purification of Myc/DDK tagged FBXO32 expressed in SKmel28 cells. Then, mass spectrometry analysis identified 216 proteins specifically associated with FBXO32 in two independent experiments (Supplementary Table 3). Analysis using David Tools identified enrichment in the GO terms associated with chromosome organization, ribosome biogenesis, RNA processing, and cellular stress (Supplementary Table 4). Consistently, among the FBXO32 partners, we found several members of chromatin remodeling complexes, including SMARCA5 belonging to the ISWI complexes [26], SMARCD1, and SMARCA4 that are associated with BAF/PBAF complexes. SMARCA4 (BRG1) was an attractive candidate to pursue since it was previously described to play a key role in melanoma biology [21]. Moreover, SMARCA4 (as well SMARCD1 and SMARCA5) could make the link between FBXO32 and the transcription machinery.

**Fig. 6:**
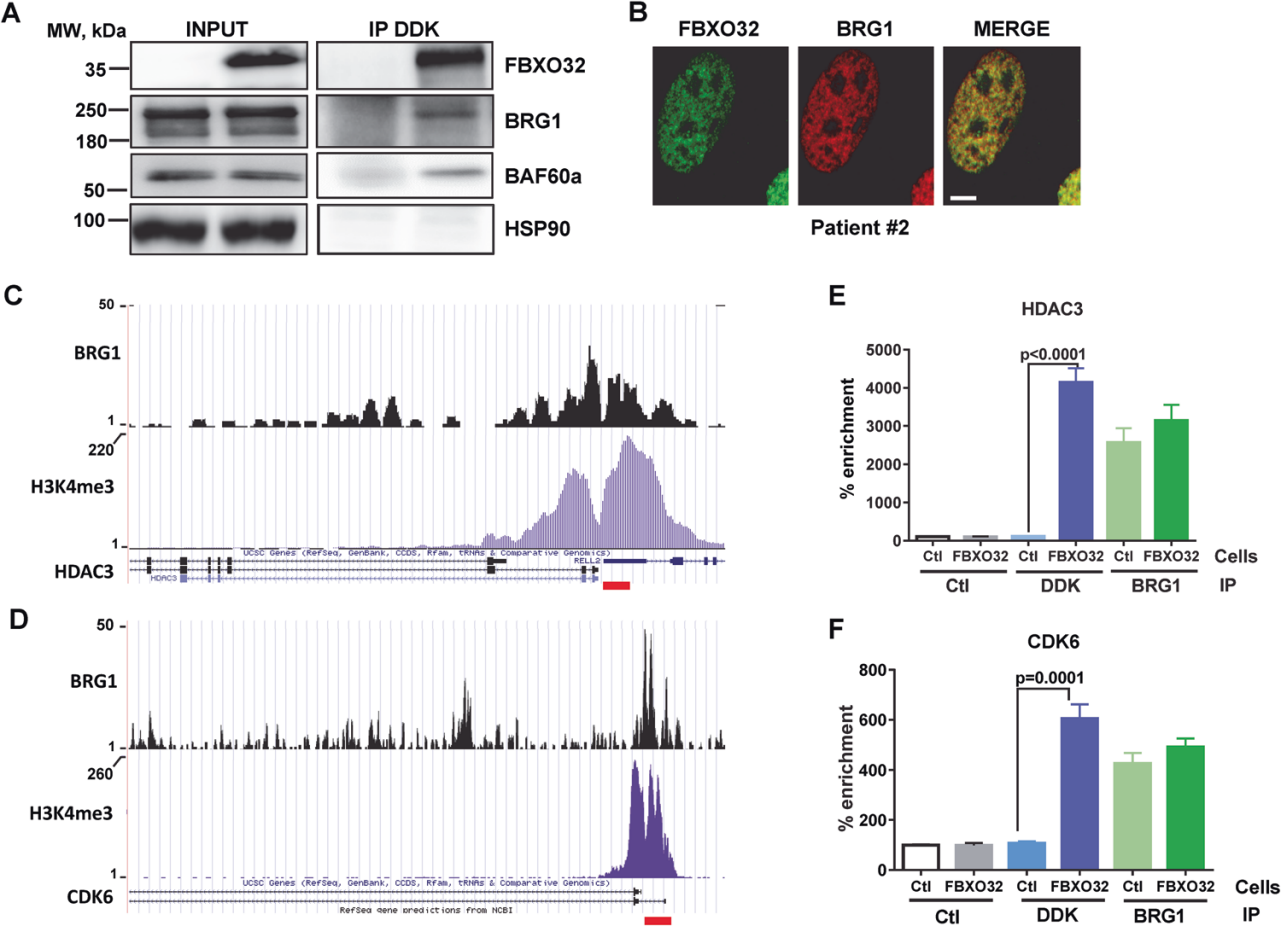
FBXO32 is associated with chromatin remodeling complex at regulated loci. **A** Co-immunoprecipitation and western blot analysis showing interaction between FBXO32 with BRG1 and BAF60a in SKmel28 transduced with Myc/DDK-tagged FBXO32 vector. **B** Immunofluorescence labeling of FBXO32 (green) and BRG1 (red) in isolated melanoma cells from patient 2. The photos taken by the confocal microscopy show a colocalization of the two proteins in the nucleus. Bar = 5 µm. **C** UCSC capture of HDAC3 locus showing BRG1-binding site (upper panel) overlapping H3K4me3 active mark (lower panel). **D** UCSC capture of CDK6 locus showing BRG1 binding site (upper panel) overlapping H3K4me3 active mark (lower panel). **E**, **F** ChIP-qPCR analysis in parental and FBXO32 overexpressing 501Mel cells, after immunoprecipitation with DDK antibody. DNA was amplified either with HDAC3 promoter primers (**E**) or CDK6 promoter primers (**F**) (mean ± SD, *n* = 3).

First, we verified the association of FBXO32 with BRG1 (*SMARCA4*). Western blot analysis after immunoprecipitation with anti-DDK showed that BRG1 was pulled down with FBXO32 in DDK-tagged FBXO32-expressing SKMEl28 cells, but not in control cells (Fig. 6A). Note that SMARCA5 (BAF60A) was also co-immunoprecipitated with FBXO32. Furthermore, we have also been able to show an interaction between endogenous BRG1 and FBXO32 in 501Mel cells after immunoprecipitation with anti-BRG1 antibody (Supplementary Fig. 7). Immunofluorescence studies showed a nuclear labeling of FBXO32 that largely overlapped with BRG1 (Fig. 6B).

Then, we hypothesized that FBXO32 interacted with BRG1 and chromatin at loci regulated upon FBXO32 silencing. First, we analyzed BRG1 [21] and H4K4me3 [4] ChIP-Seq data. We observed deposition of active histone marks at CDK6 and HDAC3 promoters, overlapping with BRG1 binding sites (Fig. 6C, D). Therefore, we performed ChIP-qPCR experiments after immunoprecipitation of chromatin either with anti-BRG1 or anti-DDK antibodies, in 501MEL cells expressing or not tagged FBXO32.

Then, qPCR analyses with primers spanning the CDK6 and HDAC3 promoter regions (red bar) showed a strong enrichment in anti-DDK immunoprecipitations from cells expressing tagged FBXO32 compared to parental cells (Fig. 6E, F). After immunoprecipitation with anti-BRG1, we also observed a strong enrichment compared to non-relevant antibody. As expected, there was no significant change between parental and DDK-FBXO32-expressing cells. These data confirm that BRG1 is bound at CDK6 and HDAC3 promoters and demonstrate that FBXO32 interacts with chromatin at loci it regulates.

## Discussion

Compelling research works demonstrated the pivotal role of MITF in melanocyte and melanoma through its ability to promote essential biological processes such as differentiation, survival, and proliferation, but also to damper motility and invasion [19]. This large pleiotropism indicates that MITF impacts and coordinates numerous key molecular pathways, to regulate melanocytes and melanoma homeostasis. Here, we demonstrate that MITF regulates global ubiquitination in melanoma cells, at least in part, through FBXO32. FBXO32 is a phosphorylation-dependent substrate recognition component of SCF E3 ligase complex and was initially identified as a key regulator in muscle homeostasis and heart development [22, 24]. However, the downregulation of FBXO32 upon MITF knockdown cannot totally explain the inhibition of nuclear ubiquitination in MITF-depleted cells. Indeed, overexpression of FBXO32 in MITF knockout cells was not sufficient to rescue the decrease in ubiquitination (data not shown). This is not surprising since MITF also regulated directly TRIM63 and indirectly many other genes involved in ubiquitination process. As no MITF binding was detected in the promoter of these genes, the said genes did not appear in our bioinformatic analysis.

More recently, FBXO32 has been also involved in cancer development and several reports ascribed an anti-tumorigenic role to FBXO32 [27, 28]. However, in agreement with our data in melanoma, FBXO32 was also shown to favor breast cancer cell xenograft development [29] and analysis of all the TCGA cohorts showed that high levels of FBXO32 were associated with bad prognosis in mesothelioma, kidney renal papillary cell carcinoma, pancreatic adenocarcinoma, and brain lower grade glioma (not shown).

The pro-tumoral function of FBXO32 was confirmed by biological studies demonstrating that FBXO32-forced expression favors proliferation and migration of melanoma cells, while FBXO32 downregulation has opposite effects. Strengthening these observations and its pro-tumorigenic role, FBXO32-forced expression greatly favors xenograft development in nude mice. The mechanisms by which FBXO32 knockdown inhibits cell proliferation is not clear. We observed no apoptosis, a weak G1 arrest, and a faint increase in senescence markers (not shown) after FBXO32 depletion.

At the molecular level, transcriptomic analysis shed light on the molecular cascades affected upon FBXO32 inhibition. FBOX32 regulates genes overlapping with that regulated by MITF, suggesting that FBXO32 might mediate some of the downstream transcriptional effects of MITF. Among the top upregulated genes, SMAD7 appears to mediate the inhibition of the TGFb pathway in FBXO32 knockdown cells, in line with previous reports showing that FBXO32 downregulation inhibits the epithelial mesenchymal transition (EMT) induced by TGFb in human breast epithelial cells [29]. Of note, the increase in the invasive properties is frequently associated with increased EMT, of cancer cells in general [30] and of melanoma cells specifically [31].

In addition, CDK6 belongs to the top downregulated genes upon FBXO32 silencing. CDK6 inhibition mimics the effect of FBXO32 knockdown, indicating that the loss of CDK6 participates to the anti-proliferative and anti-invasive effects of FBXO32 inhibition. The role of CDK6 in both proliferation and invasiveness has been already reported in squamous carcinoma cells [32]. Therefore, the transcriptional rewiring elicited by FBXO32 depletion in melanoma cells predicts an inhibition of proliferation and invasion, in agreement with the results of ours in vitro and in vivo functional studies.

To identify the link between FBXO32 and transcription regulation, we performed a proteomic analysis to identify FBXO32-interacting proteins that unveiled FBXO32 partners functioning in the chromatin remodeling process. Indeed, we identified ISWI, BAF, and PBAF components associated with FBXO32. As expected, component of the SCF complexes were also identified among FBXO32 partners. Note that FBXO32 was reported to bind and regulate CTBP1 in breast cancer cells [29], but we identified CTBP1 neither in the proteomic analyses nor in classical co-immunoprecipitation experiments (not shown).

Among the BAF components, we focused on SMARCA4, and confirmed its association with FBXO32. However, we have not been able to identify the member of the core BAF/PBAF complex that is ubiquitinated by FBXO32. We therefore concluded that FBXO32 might ubiquitinate an accessory BAF protein that in turn regulates the BAF complex activity. Nevertheless, the direct link between FBXO32, the BAF complex, and transcriptional regulation was further confirmed by ChIP-qPCR experiments showing that FBXO32 together with SMARCA4 interacted with chromatin at HDAC3 and CDK6 genes that are respectively upregulated and downregulated in FBXO32 knockdown cells.

Therefore, we hypothesized that FBXO32 can interact with both activating and repressive complexes. However, according to the proteomic results, the complexes interacting with FBXO32, BAF, PBAF, and NUMAC (containing CARM1) [33] are generally considered as activating complexes. No transcriptional repressors, such as HDAC or REST, which were reported to be associated with SMARCA4, were found in the list of FBXO32 interacting proteins. Nevertheless, it should be noted that in one of the two proteomic analyses, HDAC1 was associated with FBXO32, suggesting an interaction with the SIN3A/HDAC repressive complex [34].

In conclusion, our work establishes the role of MITF as a regulator of the ubiquitination pathway, in melanoma cells, through transcriptional regulation of several genes, among which FBXO32. FBOX32 by interacting with chromatin remodeling complexes containing SMARCA4 regulates the transcriptional repertoire and multiple key biological functions, responsible of melanoma progression and dissemination.

## Supplementary information

supplemental figures and legends

supplemental table 1

supplemental table 3
