# Supplementary material for: FBXO32 links ubiquitination to epigenetic reprograming of melanoma cells
Source: Cell Death Differ. 2021 Jan 18;28(6):1837–48. doi: 10.1038/s41418-020-00710-x (PMC8184796; doi:10.1038/s41418-020-00710-x)
Supplement: Supplementary file 1 — supplemental figures and legends [file 41418_2020_710_MOESM1_ESM.pdf]

SUPPLEMENTAL MATERIALS

FBXO32 links ubiquitination to epigenetic reprograming of melanoma cells.

Nadia Habel[1]*, Najla El-Hachem[1]*, Frédéric Soysouvanh[1]*, Hanene Hadhiri-Bzioueche[1], Serena Giuliano[1], Sophie Nguyen[1], Pavel Horák[1], Anne-Sophie Gay[2], Delphine Debayle[2], Nicolas Nottet[1], Guillaume Béranger[1], Brigitte Bressac-de Paillerets[4], Corine Bertolotto[1£] and Robert Ballotti[1£]

[1]Université Nice Côte d'Azur, Inserm U1065, C3M, Nice, France, Team 1, Biology and pathologies of melanocytes, Equipe labellisée ARC 2019. [2]CNRS, Institut de Pharmacologie Moléculaire et Cellulaire, Sophia Antipolis, France. [4]Gustave Roussy, Département de Biopathologie et INSERM U1279, Villejuif, France.

NH, NEH, FS, *equal contribution. CB and RB, [£]equal contribution. Correspondence should be addressed to Robert Ballotti: email: ballotti@unice.fr, telephone: +33 4 89 06 43 32

Running title: FBXO32 interacts with BRG1

Declaration of Interests: The authors declare no competing interests

Upstream Regulator	Molecule Type	Z-score	p-value
MITF	Transcription regulator	-2.89	1.38E-05
MYC	Transcription regulator	-2.32	2.10E-03
TGFB	Growth factor	-2.14	1.29E-02
PAX7	Transcription regulator	-1.94	2.25E-02
mir-145	Microrna	1.98	3.77E-03
TP53	Transcription regulator	2.08	1.06E-02
let-7	Microrna	2.38	3.15E-04
KDM5B	Transcription regulator	2.45	1.58E-02
miR-124	Microrna	2.69	2.45E-03

Supplemental table 2. IPA® upstream activators analysis of the genes regulated upon FBXO32 silencing.

Category	Term	Genes	Fold Enrichment	Benjamini
**Chromatin, Epigenetic**	chromosome organization	MORF4L1, MAD1L1, DYNC1LI1, NUP98, COPS6, COPS3, COPS4, HCFC1, DEK, TOP1, NCAPG, **SMARCD1**, PRIM2, H2AFY, NAT10, PRKAA1, NUP37, OGT, LRWD1, TOP2A, TRIP12, HELLS, GNL3, CKAP5, MSH2, LIG1, HMBOX1, CDK9, PKN1, RRP8, LAS1L, NUP155, SMC4, PPM1F, RECQL, PNKP, CUL4A, ASCC3, POLD2, MAPK3, MCMBP, **SMARCA5**, CLIP1, NUP107, CUL4B, AKAP8, PAF1, CARM1, **SMARCA4**, SUPT6H	2,78	7,40E-08
**Ribosome**	ribosome biogenesis	KRR1, GTPBP4, SDAD1, NOC4L, NUP88, GTPBP10, RRP8, ERAL1, LAS1L, RPL29, RSL1D1, MRPS9, BRIX1, WDR3, NAT10, NOP56, LOC102724159, UTP20, GNL1, TEX10, GNL3, NSUN4	4,32	1,48E-05
	Ribonucleoprotein complex biogenesis	KRR1, GTPBP4, SDAD1, NOC4L, CRNKL1, NUP88, GTPBP10, CNOT1, ERAL1, LAS1L, RRP8, RPL29, RSL1D1, MRPS9, BRIX1, WDR3, CELF2, NAT10, NOP56, LOC102724159, UTP20, GNL1, EIF3M, TEX10, NSUN4, GNL3	3,57	2,17E-05
**Cell Cycle**	mitotic cell cycle process	DYNC1LI1, MAD1L1, NUP98, USP8, CNOT1, TUBGCP3, NUMA1, NCAPG, PRIM2, H2AFY, PRKACA, NUP37, TOP2A, HELLS, TUBB3, CEP131, NUP88, CKAP5, MSH2, LIG1, BIRC6, NUP155, DCTN1, SMC4, NAE1, PSMC4, CUL4A, PSMD12, FGFR1OP, MCMBP, CLIP1, CDK11B, NUP107, CUL4B, AKAP8, CARM1, GIGYF2, SMARCA4, DNM2	2,89	2,94E-06
	mitotic cell cycle	DYNC1LI1, MAD1L1, NUP98, USP8, ASNS, CNOT1, TUBGCP3, NUMA1, PAK2, NCAPG, PRIM2, H2AFY, PRKACA, NUP37, TOP2A, HELLS, TUBB3, CEP131, NUP88, CKAP5, MSH2, LIG1, BIRC6, NUP155, DCTN1, SMC4, NAE1, PSMC4, CUL4A, PSMD12, FGFR1OP, MCMBP, CLIP1, CDK11B, NUP107, CUL4B, AKAP8, CARM1, GIGYF2, SMARCA4, DNM2	2,79	3,16E-06
	cell cycle	MAD1L1, DYNC1LI1, TSG101, PRKAG1, CNOT1, PAK2, PRIM2, H2AFY, NUP37, PRKACA, TOP2A, TUBB3, DHCR24, GTPBP4, NUP88, LIG1, HERC5, CDK9, RRP8, DCTN1, FGFR1OP, MAPK3, MCMBP, CLIP1, CDK11B, AKAP8, NUP107, CARM1, GIGYF2, SMARCA4, NUP98, USP8, HCFC1, ASNS, TUBGCP3, NUMA1, NCAPG, SH3GLB1, PRKAA1, HELLS, CEP131, MSH2, CKAP5, BIRC6, NUP155, APPL1, SEPT10, SEPT11, SMC4, NAE1, RPS6KA3, PSMC4, PSMD12, CUL4A, CUL4B, DNM2	2,21	8,53E-06
	cell cycle process	DYNC1LI1, MAD1L1, NUP98, USP8, TSG101, PRKAG1, CNOT1, TUBGCP3, NUMA1, SH3GLB1, NCAPG, PRIM2, H2AFY, PRKAA1, PRKACA, NUP37, TOP2A, HELLS, TUBB3, DHCR24, CEP131, NUP88, CKAP5, MSH2, LIG1, BIRC6, CDK9, RRP8, NUP155, DCTN1, SMC4, NAE1, PSMD12, PSMC4, CUL4A, FGFR1OP, MCMBP, CLIP1, CDK11B, NUP107, AKAP8, CUL4B, CARM1, GIGYF2, SMARCA4, DNM2	2,23	1,03E-04
**RNA**	ncRNA metabolic process	ELAC2, PUS1, UBA5, TRMT1, POLR2A, WARS, H2AFY, NAT10, MRPL39, GTF3C1, NSUN4, KRR1, IKBKAP, GTPBP4, MOCS3, NOC4L, CDK9, RRP8, LAS1L, METTL2B, RPL29, RSL1D1, MRPS9, POP1, FARSB, WDR3, FARSA, NOP56, LOC102724159, UTP20, CPSF1, TEX10, SMARCA4	3,81	1,14E-07
	RNA processing	ELAC2, PUS1, CRNKL1, TRMT2A, U2SURP, TRMT1, UBA5, IVNS1ABP, POLR2A, DCPS, HNRNPM, GPKOW, PRKACA, NAT10, NSUN4, KRR1, IKBKAP, GTPBP4, MOCS3, CSTF3, NOC4L, CDK9, LAS1L, RRP8, METTL2B, RPL29, RSL1D1, MRPS9, PPP1R8, CPSF7, POP1, WDR3, CELF2, PAF1, NOP56, LOC102724159, UTP20, CSTF1, CPSF1, TEX10, SUPT6H	2,97	6,33E-07
	ncRNA processing	KRR1, IKBKAP, GTPBP4, MOCS3, ELAC2, NOC4L, PUS1, RRP8, UBA5, TRMT1, LAS1L, METTL2B, RPL29, RSL1D1, MRPS9, POP1, WDR3, NAT10, NOP56, LOC102724159, UTP20, CPSF1, TEX10, NSUN4	3,85	2,21E-05
**Cellular Stress**	cellular response to stress	ARMT1, MORF4L1, MRPS35, COPS6, CLPB, COPS3, COPS4, DEK, CNOT1, USP10, NUP37, TOP2A, GNL1, EIF2B3, AUP1, NUP88, LIG1, ERLIN2, PKN1, CDK9, RRP8, DCTN1, RSL1D1, PNKP, RECQL, SERPINB6, MRPS9, ASCC3, MAPK3, UCHL5, SMARCA5, NUP107, CARM1, GIGYF2, NUP98, HACD3, UBA5, ASNS, POLR2A, STT3B, SH3GLB1, INPP5F, PRKAA1, SEC61A1, TRIP12, NPLOC4, MSH2, NUP155, RPS6KA3, PSMC4, CUL4A, GSK3A, GFPT1, POLD2, FAF2, CUL4B, DNM2	2,12	2,04E-05

Supplemental table 4. GO terms enrichment associated with the proteins interacting with FBXO32

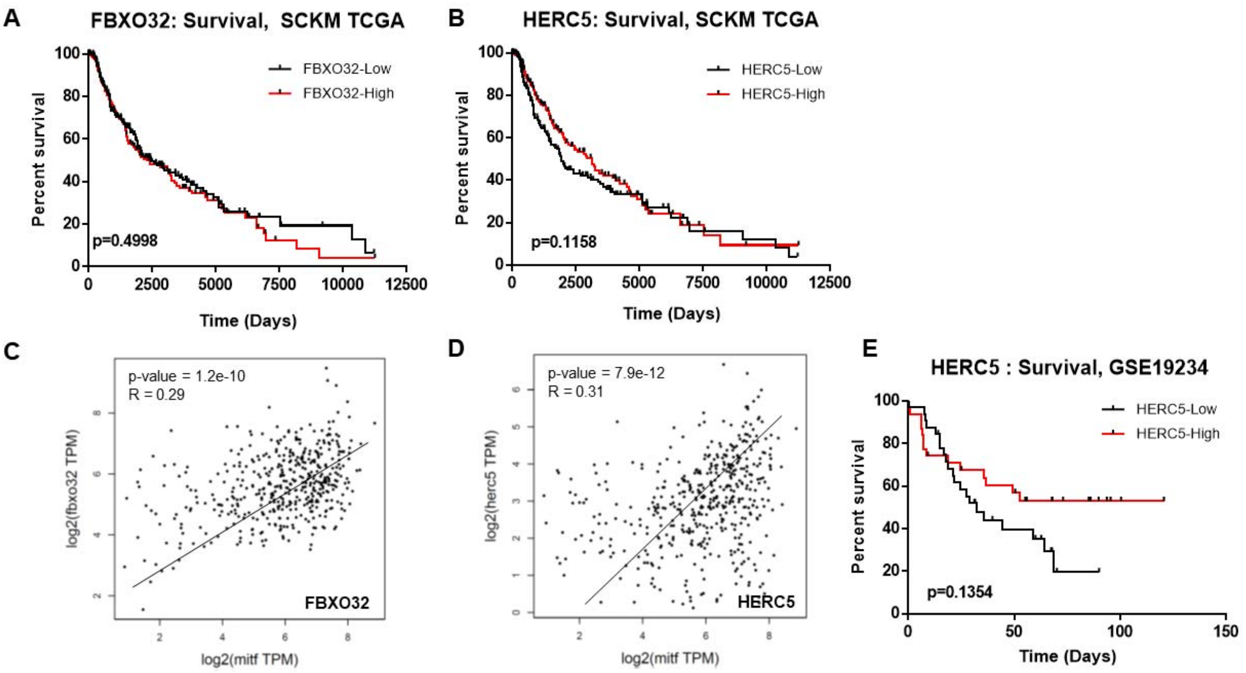

Supplemental Figure 1. (A) Kaplan-Meier survival curve of cutaneous melanoma patients with low (black line) or high (red line) expression level of FBXO32 from TCGA SKCM dataset. (B) Survival curve of melanoma patients with low (black line) or high (red line) expression level of HERC5 from TCGA SCKM dataset. (C) Correlation between MITF and FBXO32 expression in melanoma from TCGA cohort. (D) Correlation between MITF and HERC5 expression in melanoma from TCGA cohort. (E) Survival curve of melanoma patients with low (black line) or high (red line) expression level of HERC5 from GSE19234 dataset.

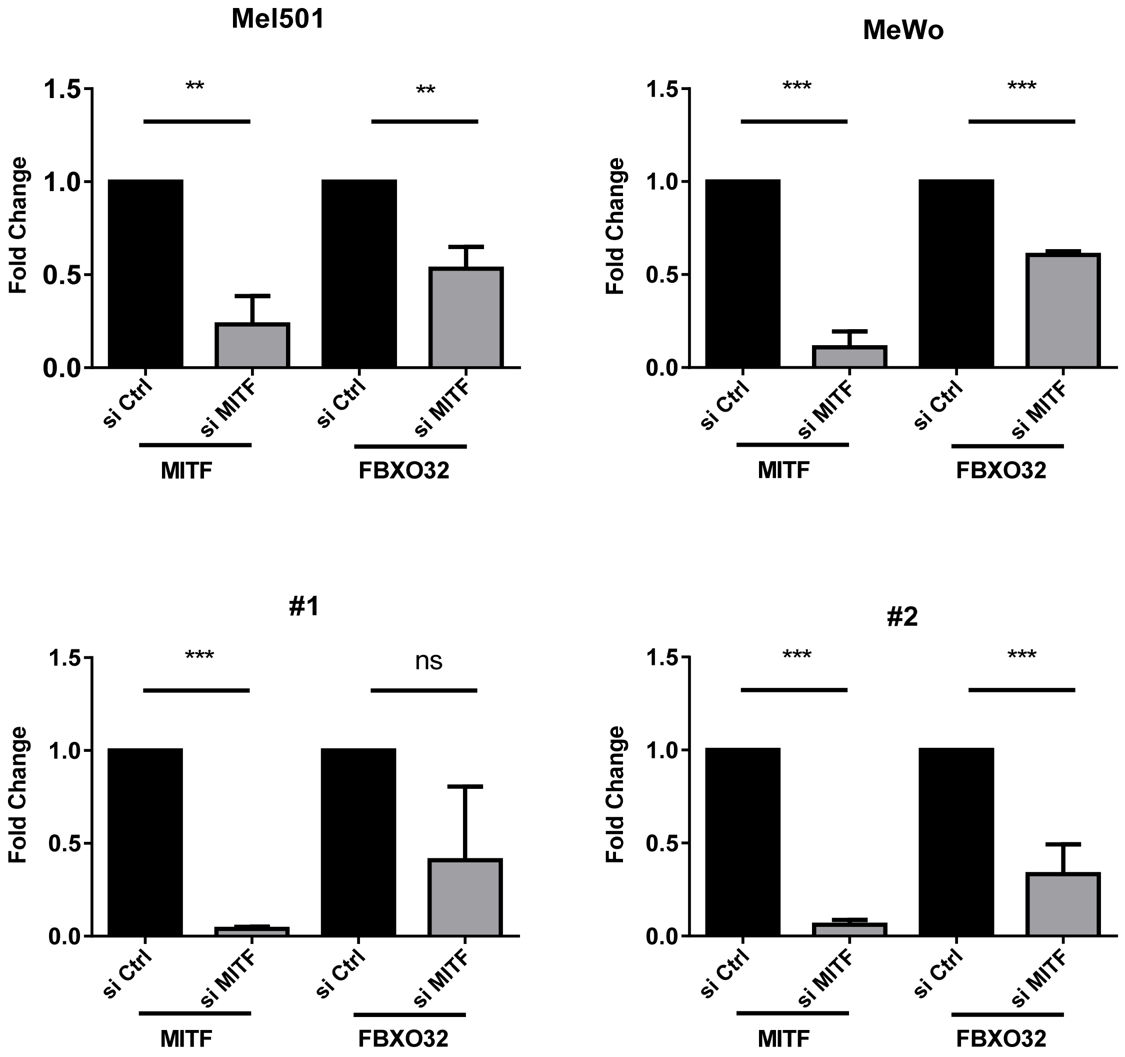

Supplemental Figure 2. Relative expression of MITF and FBXO32 when MITF is downregulated by siRNA in (A) 501Mel cells, (B) MeWo cells, and (C, D) short-term culture isolated from patients. *: p-value < 0.05, **: p-value < 0.01, ***: p-value < 0.001.

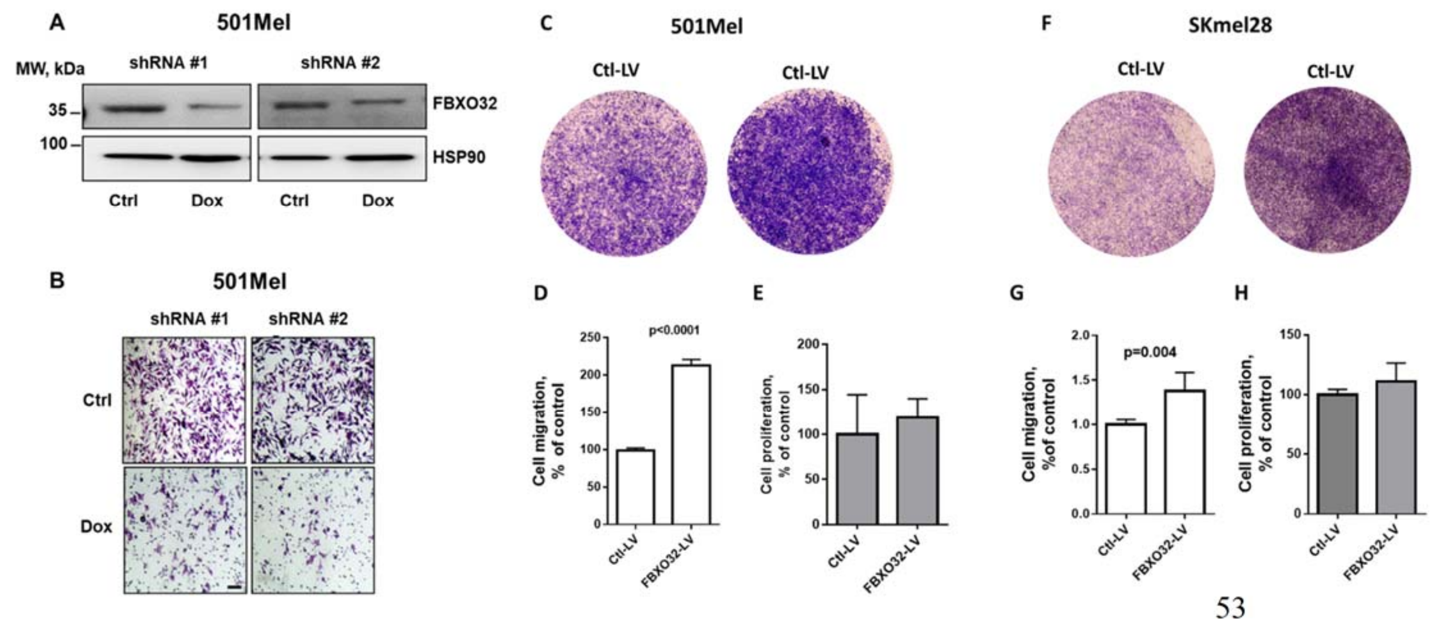

Supplemental Figure 3. (A) Expression of FBXO32 in 501Mel after transduction with doxycycline inducible shRNAs targeting FBXO32. HSP90 was probed as loading control. (B) Effects of shRNAs-mediated FBXO32 downregulation on migration of 501Mel cells. Bar=100 µm. (C) Expression of FBXO32 in 501Mel after transduction with empty or FBXO32 lentivirus. HSP90 was probed as loading control. (D, E) Effects of FBXO32 overexpression, on migration of 501Mel cells (D) representative image, (E) quantification of 3 independent experiments (Mean+/- SD). (F) Effects of FBXO32 overexpression on proliferation of 501Mel cells. Graph represent mean+/-SD of 3 independent experiments. (G) Expression of FBXO32 in SKmel28 after transduction with empty or FBXO32 lentivirus. HSP90 was probed as loading control. (D, E) Effects of FBXO32 overexpression, on migration of SK28mel cells (D) representative image, (E) quantification of 3 independent experiments (Mean+/- SD). (F) Effects of FBXO32 overexpression on proliferation of SKmel28 cells. Graph represent mean+/-SD of 3 independent experiments.

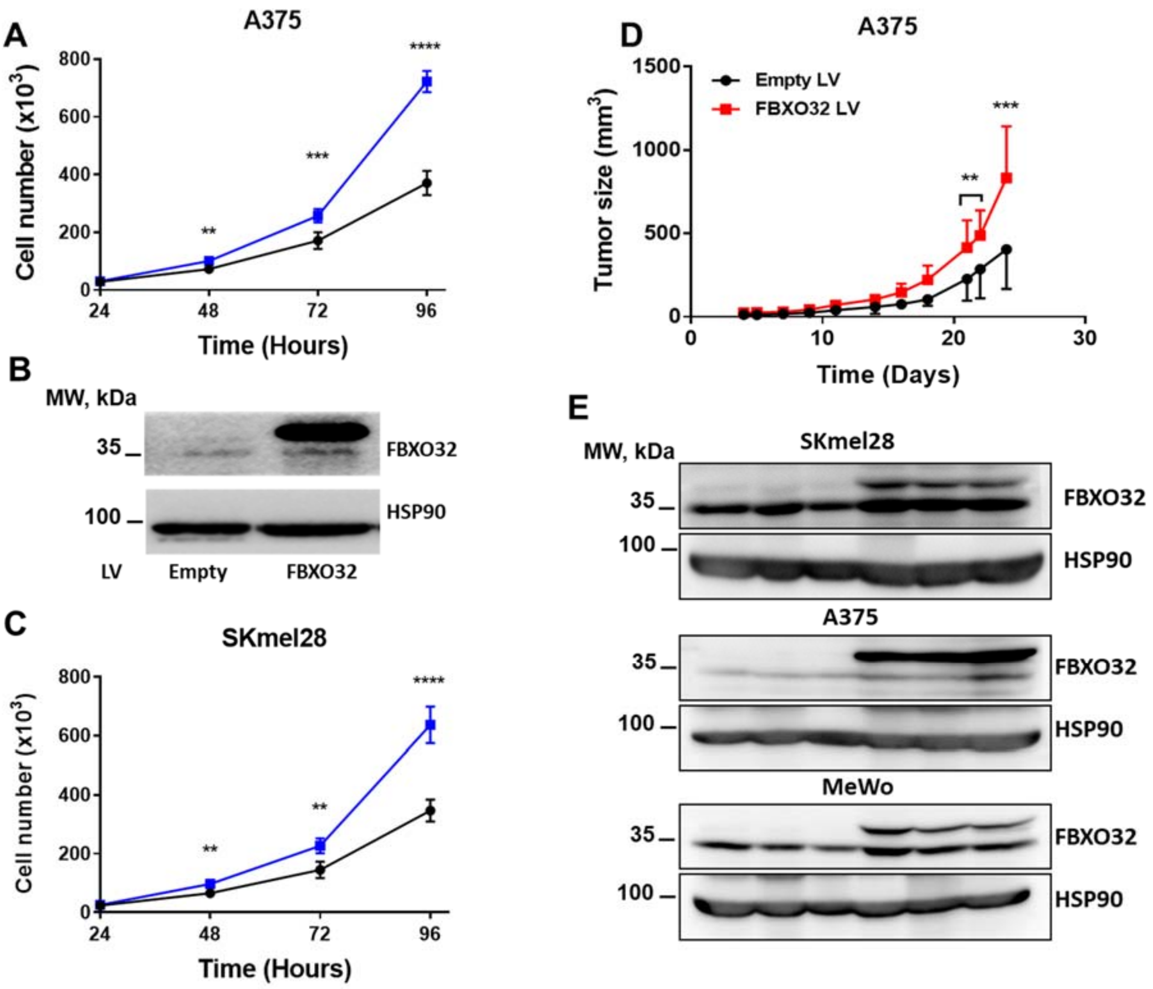

Supplemental Figure 4. (A) Quantification of A375 cells proliferation after empty vector (black line) or FBXO32 virus (blue line) transduction, from 24h to 96h (mean ± SD, n = 6). (B) Western blot analysis of FBXO32 expression after lentivirus mediated FBXO32 forced expression in A375 cells. HSP90 expression was probed as loading control. (C) Quantification of SKmel28 cells proliferation after empty vector (black line) or FBXO32 (blue line) transduction, from 24h to 96h (mean ± SD, n = 6). (D) Tumor growth after A375 cells xenografts transduced with empty vector (black line) or FBXO32 (red line) (mean ± SD, n = 10). (E) Expression of FBXO32 in 6 different tumors after SKmel28, A375 or MeWo cells xenografts. HSP90 was probed as loading control.

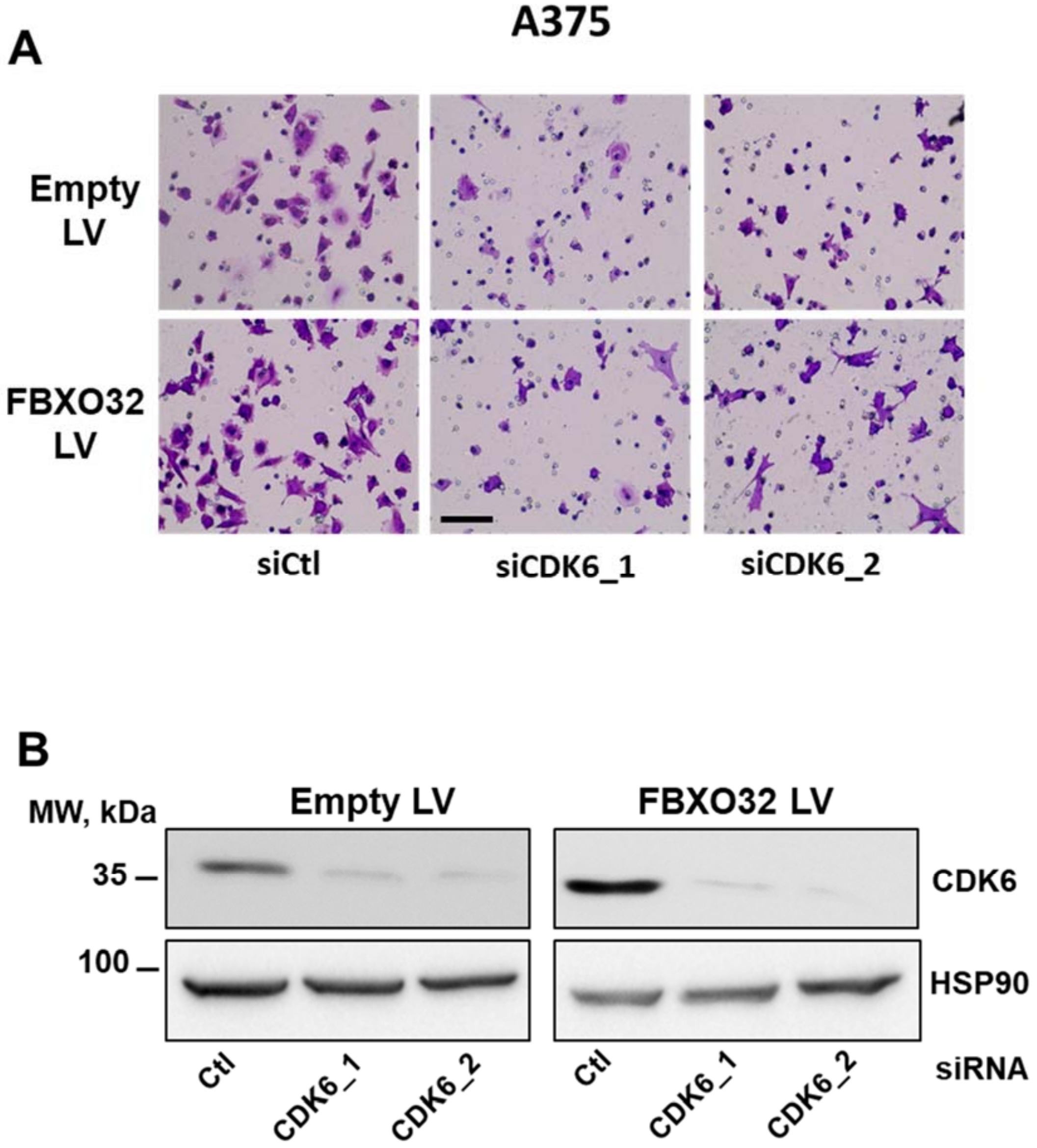

Supplemental Figure 5. (A) Representative image of the effect of CDK6 downregulation on migration of A375 overexpressing (lower panel) or not (upper panel) FBXO32. Bar=100 µm. (B) Western blot analysis of the expression of CDK6 after siRNAs downregulation in A375 cell with or without FBXO32 forced expression. HSP90 was probed as loading control.

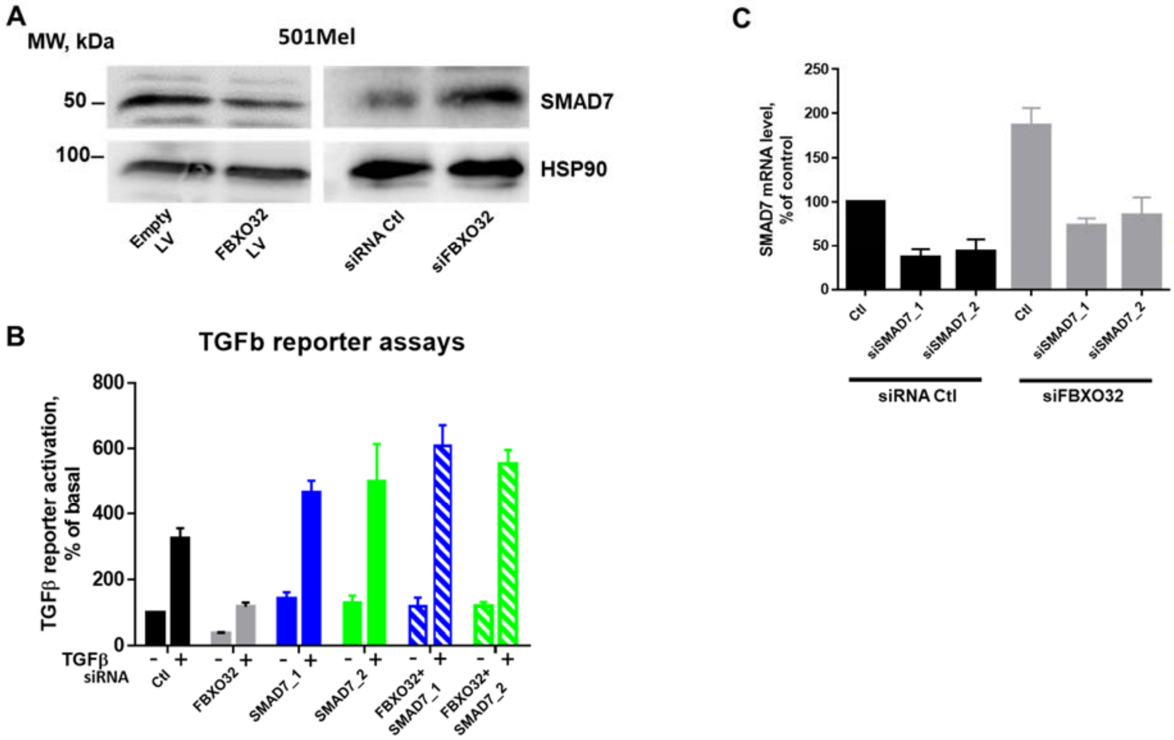

Supplemental Figure 6. (A) Western blot analysis of SMAD7 expression after transfection of siRNAs targeting FBXO32 (right panel) or transduction by FBXO32 expressing lentivirus (left panel) in 501Mel cells. HSP90 was probed as loading control. (B) 501Mel were transfected with the TGFb reporter and the indicated siRNAs. When indicated cell were incubated to TGFb (5nM, for 18h). TGFb reporter activity was calculated as % of basal activity (mean ± SD, n = 3). (C) qPCR analysis of SMAD7 expression in the different conditions as in B.

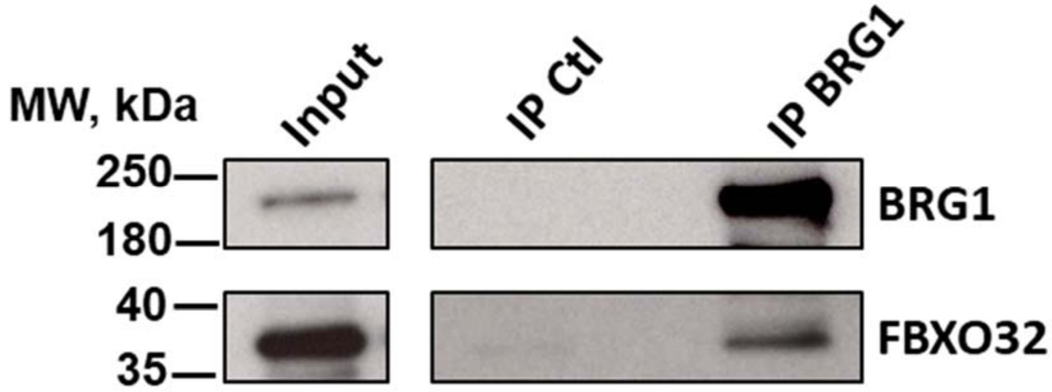

Supplemental Figure 7. Co-immunoprecipitation and western blot analysis showing interaction between FBXO32 with BRG1 in 501Mel cells. Western blot with anti-BRG1 and anti-FBXO32 in total extract (Input), in immune-precipitates with control (IP Ctl) or BRG1 (IP BRG1) antibodies.
